# Supplementary material for: Tead1a Initiates Transcriptional Priming Through the TEAD1a/YAP‐Notch1‐Spi1/Cebpα Axis to Promote Neutrophil Fate
Source: Adv Sci (Weinh). 2025 Aug 28;12(41):e05441. doi: 10.1002/advs.202505441 (PMC12591127; doi:10.1002/advs.202505441)
Supplement: Supplementary file 4 — Supplementary Dataset [file ADVS-12-e05441-s004.zip › 3-7.zip!/3-7/02-7.cluster/cluster19/Enrich/ego_kegg.pdf]

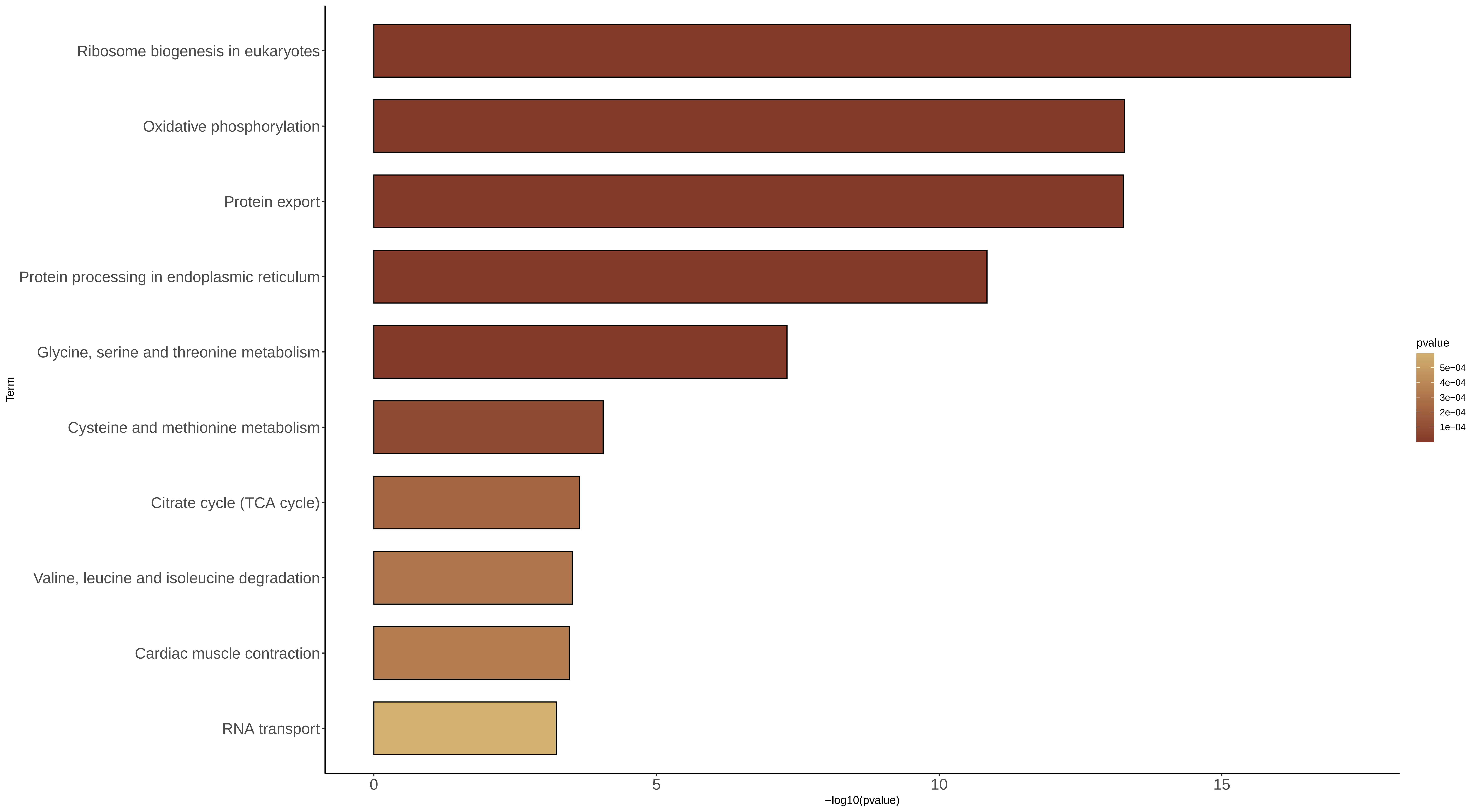

Ribosome biogenesis in eukaryotes
Oxidative phosphorylation
Protein export
Protein processing in endoplasmic reticulum
Glycine, serine and threonine metabolism
Cysteine and methionine metabolism
Citrate cycle (TCA cycle)
Valine, leucine and isoleucine degradation
Cardiac muscle contraction
RNA transport
Term
0
5
10
15
−log10(pvalue)
pvalue
5e−04
4e−04
3e−04
2e−04
1e−04
